# Abrupt onset of tongue deformation and phase space response of ions in magnetically-confined plasmas

**DOI:** 10.1038/srep36217

**Published:** 2016-10-31

**Authors:** K. Ida, T. Kobayashi, K. Itoh, M. Yoshinuma, T. Tokuzawa, T. Akiyama, C. Moon, H. Tsuchiya, S. Inagaki, S.-I. Itoh

**Affiliations:** 1 National Institute for Fusion Science, National Institutes of Natural Sciences, Toki, 509-5292, Japan; 2 SOKENDAI (The Graduate University for Advanced Studies), 322-6 Oroshi, Toki, Gifu 509-5292, Japan; 3 Research Institute for Applied Mechanics, Kyushu Univ., Kasuga, 816-8580, Japan

## Abstract

An abrupt onset of the new tongue-shaped deformation of magnetic surface in magnetized plasmas, which was conjectured in since the 1960s but has not been observed, is experimentally identified just before an abrupt onset of a large-scale collapse event. Two novel properties of the event are identified. First, the transition of symmetry of perturbation (rather than a growth of linearly unstable MHD modes) was found to be a key for the onset of abrupt collapse, i.e., the transition of symmetry gives a new route to the collapse from stable state. Second, as a phase-space response of ions, the distortion from Maxwell-Boltzmann distribution of epithermal ions was observed for the first time.

Abrupt onset of violent activities has been widely observed in plasmas, e.g., solar flare^1^, disruptions and edge-localized-modes^2^ in confinement devices^3^. The key issue here is the trigger problem^4^,^5^,^6^, i.e., the growth rate of deformation jumps to a large value while there is almost no change in parameters that control linear stability. Nonlinear models have been proposed^7^,^8^,^9^, but the mechanism remains unclear. In magnetized plasma, there are various MHD modes due to the instabilities. The MHD models based on the linear instability have been developed, but they fail to model the MHD collapse event^10^. The mechanism of the MHD mode stabilization and destabilization are studied based on the critical gradient or resonance conditions of the plasma parameters. The magnetic field structure of the MHD instability has been considered to have poloidal (*m*) and toroidal (*n*) mode numbers as a resonance. Although the non-resonant structure of magnetic field perturbation, namely, ‘tongue’ structure, was predicted theoretically by Arstimovich^11^, it has not been observed in toroidal plasmas. Only the observation of the finger-like structure^12^ at the ELM crash implies the existence of non-resonant mode. In the high temperature plasma, the velocity distribution of bulk ions can be destroyed by the MHD instability. However, the impact on velocity distribution of bulk ions (distortion of Maxwell-Boltzmann distribution) has not been studied in spite of its importance.

Here we report the discovery of the abrupt onset of the perturbation with the tongue-shaped topology (localized in poloidal and toroidal directions) which leads to the sudden collapse of electron temperature and the deformation of phase-space distribution of carbon ions in toroidal plasmas. The path of the onset, i.e., the transition from mirror symmetric ‘tongue’ to helical symmetric mode with long wavelength along the magnetic field is identified. The trigger problem, which is a long-lasting mystery, may be resolved by studying the dynamical evolution of tongue-like (non-mode) structure. The observation of the ‘tongue’ deformation is a clue for understanding the mechanism of the trigger problem in plasmas.

## Results

### Observation of magnetic field tongue event

**Figure 1:**
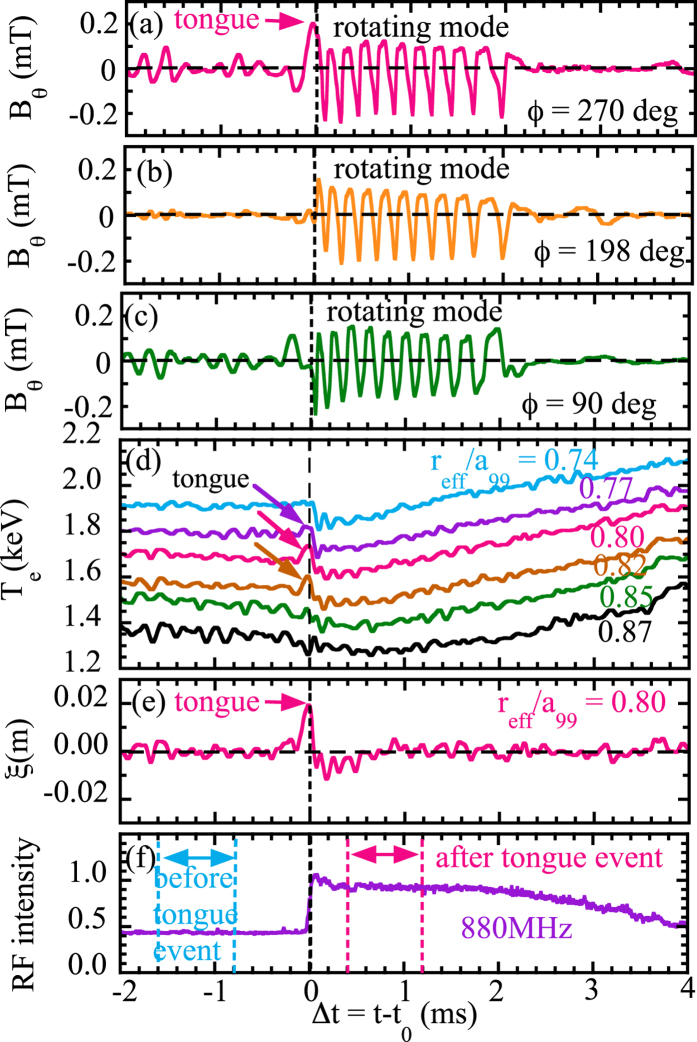
Time evolution of magnetic field *B*_*θ*_ at toroidal angle *ϕ* of (**a**) 270°, (**b**) 198°, (**c**) 90°, (**d**) electron temperature measured with electron cyclotron emission (ECE) radiometer (*ϕ* = 198°) at *r*_eff_/*a*_99_ = 0.74, 0.77, 0.80, 0.82, 0.85, 0.87, (**e**) displacement of the contour of electron temperature at *r*_eff_/*a*_99_ = 0.80, and (**f**) RF intensity measured with RF radiation probe (*ϕ* = 121°). Here *t*_0_ is the time when the RF radiation probe signal jumps and is 4.71573 sec. There are two perpendicular neutral beams with the beam energy of 40 keV at *ϕ* = 306° and 50 keV at *ϕ* = 90°, respectively.

Figure 1 shows time evolution of the plasma parameters at the typical ‘tongue’ event in Large Helical Device^13^,^14^. The magnetic field perturbation at the toroidal angle *ϕ* of 270°, where the magnetic probes^15^ locate near the NBI port (36° apart from the NBI port), abruptly starts to increase 130 *μ*s before the rapid increase of the RF signal (Δ*t* = 0). Then MHD oscillations with the frequency of 5–10 kHz starts and the frequency decreases in time. There is no magnetic field perturbation observed at the toroidal angle of 198° (108° apart from NBI port), until the MHD oscillations start. The symmetry of abruptly growing tongue-shaped magnetic field perturbation is quite different from that of the following MHD oscillations (Δ*t* > 0) for which the poloidal/toroidal mode numbers (*m*, *n*) of the MHD oscillations is (1, 1). The ‘tongue’ event is characterized by the abrupt increase of magnetic field perturbation toroidally localized (only at *ϕ* = 270°), which is different from the out-of-phase perturbations at *ϕ* = 90° and 270° observed well before the event (−2 ms < Δ*t* < 0.2 ms) and the rotating MHD oscillations after the event (Δ*t* > 0). After the ‘tongue’ deformation, the collapse of electron temperature and the transition from mirror symmetry across the surface *ϕ* = 90° and 270° to helical symmetry with *n*/*m* = 1/1 mode occur. The rotating MHD oscillation starts 40 *μ*s after the displacement of plasma becomes maximum.

The displacement of plasma, *ξ*, in this eruption can be evaluated from the time evolutions of electron temperature and its gradient measured with electron cyclotron emission (ECE)^16^ (Fig. 1e). The outward displacement of the contour of electron temperature is peaked at *r*_eff_/*a*_99_ = 0.8 and it starts to increase 100 *μ*s before the MHD oscillations. The displacement increases up to 2 cm and the radial gradient of the displacement, ∂*ξ*/∂*R*, at *r*_eff_/*a*_99_ = 0.85 reaches up to 0.6 at the time of onset of RF radiation probe signal. After the large displacement of plasma as indicated in Fig. 1e inside the plasma, the perturbation of the magnetic field starts to rotate in both toroidal and poloidal directions for Δ*t* > 0. The displacement decays in a few hundred *μ* sec and becomes less than a few mm during the MHD oscillation (0.2 < Δ*t* < 2 ms). The abrupt increase of RF intensity measured with RF radiation probes^17^ seen in Fig. 1f indicates the rapid increase of ion cyclotron emission (ICE) at high order harmonic frequency due to the reversal of energy distribution of the energetic ion injected by the neutral beam^18^,^19^,^20^.

**Figure 2:**
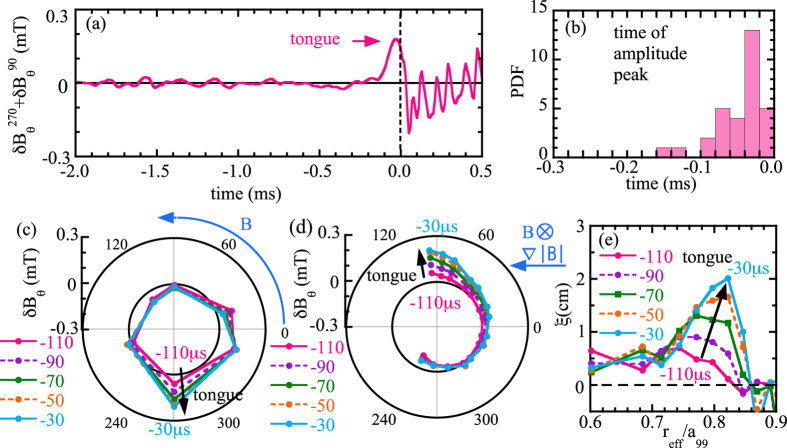
Time evolution of sum of magnetic field *B*_*θ*_ at *ϕ* = 90 and 270° in toroidal array and (**b**) probability distribution function of time of its amplitude peak and polar plot of magnetic field perturbation *B*_*θ*_ of (**c**) toroidal array and (**d**) poloidal array and (**e**) radial profiles of the plasma displacement during the ‘tongue’ event (*t* = −110, −90, −70, −50 and −30 *μ* sec).

Since the symmetry transition occurs in the event at Δ*t* ~ 0, the structure of the magnetic field perturbation is investigated by plotting the toroidal and poloidal distribution of the perturbation field rather than the conventional ‘mode’ analysis. Here, the time evolution of a sum of the magnetic probe signal at *ϕ* = 90° and 270° is plotted in order to distinguish the ‘tongue’ deformation from the out-of-phase perturbations. As seen in Fig. 2a, the ‘tongue’ deformation appears ~100 *μ*s before the abrupt increase of RF intensity. Figure 2(b) is a probability distribution function of the time of the amplitude peak of the sum of the magnetic probe signal. The ‘tongue’ deformation appears at all the events with a toroidally and poloidally localized large magnetic field perturbation of > 0.1 mT, which is larger than the out-of-phase perturbations before. The formation of the ‘tongue’ becomes largest 30 *μ*s before the abrupt increase of RF intensity and the collapse of electron temperature.

As seen in Fig. 2c,d, the perturbation of the magnetic field is localized near the NBI port in toroidal angle (*ϕ* = 270°) and in the direction of *B* × ∇*B* drift of ion in poloidal angle (*θ* = 90°), which clearly indicates the ‘tongue’ characteristics of magnetic field perturbation different from that of usual MHD modes. As seen in Fig. 2e, the displacement of the plasma is also localized radially at *r*_eff_/*a*_99_ ~ 0.8, which is further inside the location of *ι*/(2*π*) = 1 rational surface at *r*_eff_/*a*_99_ ~ 0.9, where the *m*/*n* = 1/1 MHD oscillations are localized. It is clearly demonstrated that this abrupt event occurs with the tongue-shaped topology in a short time (−100 *μ*s < Δ*t* < 0) and then the perturbation evolves into a rotating *m*/*n* = 1/1 mode. This clear localization of the perturbation field before the MHD oscillation indicates that this MHD oscillation is triggered by the MHD ‘tongue’ event rather than the growth of the MHD mode. This observation is also consistent with the fact that there is a delay of 30 *μ*s between the start of the oscillation of the magnetic probe signal (rotation of the MHD mode) and the maximum of the displacement of plasma at *r*_eff_/*a*_99_ = 0.8 detected by ECE signals.

### Distortion of Maxwell-Boltzmann distribution of ions

**Figure 3:**
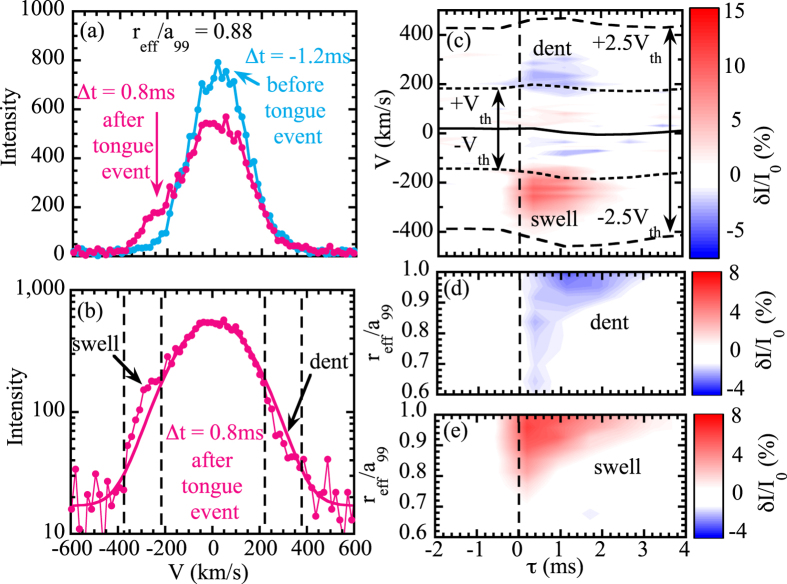
(**a**) Velocity distribution of carbon impurity before (Δ*t*_0_ = −1.2 ms) and after (Δ*t*_0_ = 0.8 ms) the ‘tongue’ event, (**b**) velocity distribution and fitted Gaussian profile after the ‘tongue’ event at *r*_eff_/*a*_99_ = 0.88 (*R* = 4.354 m). The time windows for the measurements of velocity distribution are indicated in Fig. 1. Contour of (**c**) the magnitude of velocity distortion from Maxwell-Boltzmann distribution in time and velocity at *r*_eff_/*a*_99_ = 0.88, where the distortion is most significantly observed and the magnitude of the (**d**) ‘dent’ and (**e**) ‘swell’ integrated from *v*_th_ to 2.5 *v*_th_ velocity and from −*v*_th_ to −2.5 *v*_th_ velocity in time and space normalized by the peak intensity *I*_0_.

The charge exchange spectroscopy is a standard tool to measure the radial profiles of ion temperature and toroidal rotation velocity of carbon impurity^21^,^22^. Here the charge exchange spectroscopy is used to investigate how the ion velocity distribution in the plasma changes associated with the ‘tongue’ event. Figure 3a,b show the time evolution of magnetic field perturbation and RF radiation probe signal to indicate the time window of the charge exchange spectroscopy with the integration time of 0.8 ms. There are clear differences in the velocity distribution of ion 1.2 ms before and 0.8 ms after the onset of MHD oscillation triggered by ‘tongue’ event. The clear increase of ion population is observed in the velocity range of −400 to −200 km/s. Here the sign of the velocity is positive for co-traveling ions and minus for counter traveling ions. This change is due to the distortion of Maxwell-Boltzmann distribution of ions, which is more visible in the plot of comparison of measured ion distribution with the fitted gaussian profile (solid line in Fig. 3b. Here the region where the ion population increases is called ‘swell’, while the region where the ion population decreases is called ‘dent’ in this paper. The ‘swell’ and ‘dent’ structure are observed in the velocity range of −400 to −200 km/s and 200 to 400 km/s, respectively.

Although the integration time of charge exchange spectroscopy is 0.8 ms, fast response in respect to the reference time of *t*_0_ can be studied by using conditioning reconstruction technique^23^. This distortion of Maxwell-Boltzmann distribution of ions appears transiently in the time scale of ion-ion collision time (a few ms) just after the magnetic field ‘tongue’ event as seen in Fig. 3c–e. Here the x-axis is the relative time difference between the onset of ‘tongue’ event and measurements of velocity distribution by charge exchange spectroscopy (central time of integration time window of the measurements). Here *t*_0_ is the time when the RF radiation probe signal jumps and conditional reconstruction for all ‘tongue’ events in one discharge are performed. The velocity range of the distortion is from *v*_th_ to 2.5 *v*_th_, where *v*_th_ is thermal velocity of carbon ions for both the ‘swell’ and the ‘dent’ distortions. The distortion from Maxwell-Boltzmann distribution is observed in the outer half of the plasma at *r*_eff_/*a*_99_ > 0.6~0.7. The time duration of the distortion is less than ~1 ms at *r*_eff_/*a*_99_ = 0.80, where the displacement of plasma due to ‘tongue’ has maximum and becomes longer (3~4 ms) near the plasma edge.

The distortion from the Maxwell-Boltzmann is characterized by the simultaneous decrease of co-traveling and increase of counter-traveling ions (‘dent’ and ‘swell’) in outer region of the magnetic field tongue. The ‘dent’ and ‘swell’ distortion is symmetric in the velocity space and appears in the same region in the plasma minor radius. This magnetic field tongue is observed in the low density discharge with perpendicular neutral beam injection, where a significant perpendicular pressure gradient by trapped ions exists in the plasma. This fact indicates that the distortion is due to the change in trapped ions rather than the passing particle, and decrease of co-traveling (increase of counter-traveling) is due to the abrupt flattening of trapped ions with the parallel velocity of *v*_th_ to 2.5 *v*_th_. This is clear evidence for the distortion of epithermal ions in the plasma triggered by the magnetic field ‘tongue’ event.

### Change in the radial electric field and turbulence at the ‘tongue’ event

In this section the mechanism for the distortion of epithermal ions is commented upon. Because the ion flow in the co-direction is generated by the density gradient of trapped ions, simultaneous increase/decrease of counter-/co-traveling particles observed shows the decrease of trapped ion density gradient due to the transient outward drift of trapped ions associated with the abrupt change in radial electric field^24^.

**Figure 4:**
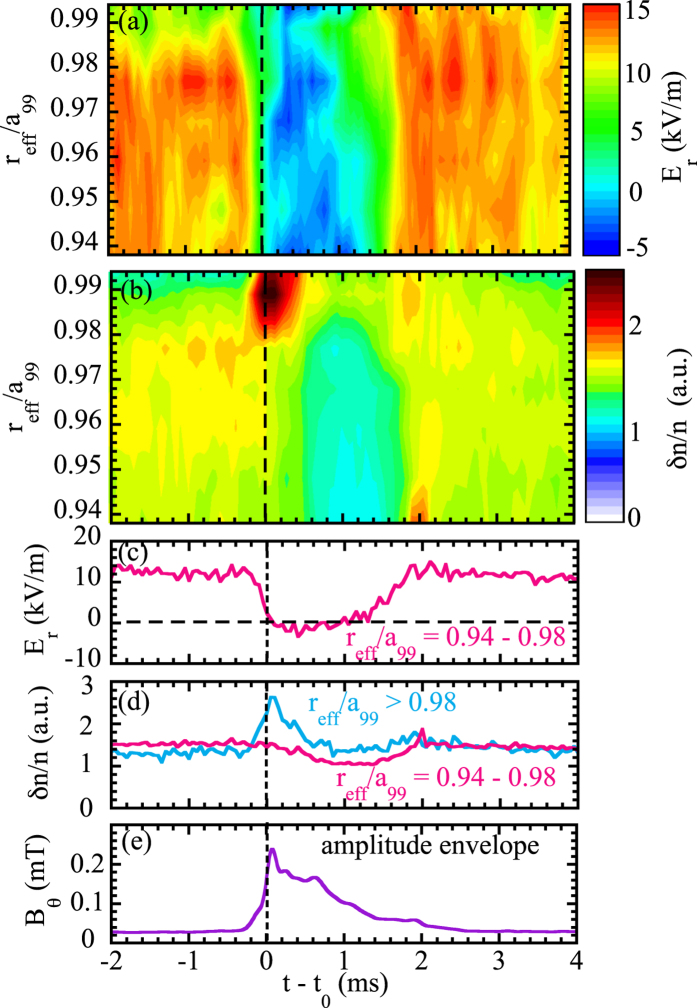
Contour of (**a**) radial electric field and (**b**) turbulence amplitude in the frequency range of 150–500 kHz in time and space using conditioning accumulation measured with Doppler reflectometer and time evolution of (**c**) radial electric field and (**d**) turbulence amplitude, and (**e**) the envelop of magnetic field burst oscillations.

There are two experimental evidences to support the outward drift of trapped ions. First, the rapid increase of RF magnetic signal strongly suggests that the hot ions in the plasma core drift to the region of cold ions near the plasma edge after the ‘tongue’ event. Second is the rapid change in mean radial electric field from large positive of 13 kV/m to small negative of −2 kV/m near the plasma edge (*r*_eff_/*a*_99_ = 0.94–0.98), measured with Doppler reflectometer^25^ as seen in Fig. 4. The x-axis is the time difference between the time of the Doppler reflectometer measurements and the magnetic field ‘tongue’ event, while the y-axis is the normalized averaged minor radius determined by the density profiles measured with multi-channel FIR interferometer and the frequency of the Doppler reflectometer at ‘tongue’ events in one discharge, where the electron density slightly decreases in time. As the electron density decreases, the location of the Doppler reflectometer measurements moves inward from *r*_eff_/*a*_99_ = 0.994 to 0.938.

When the radial electric field recovers at 2 ms after the ‘tongue’ event, both the reversal of ion energy distribution at the edge and the distortion of epithermal ions start to disappear in the ion-ion collision time scale. The turbulence in the frequency range of 150–500 kHz also shows an interesting change at the ‘tongue’ events. Before the ‘tongue’ event, the relatively large turbulence amplitude is observed near the plasma edge at *r*_eff_/*a*_99_ = 0.94–0.98. After the ‘tongue’ event, turbulence amplitude at *r*_eff_/*a*_99_ = 0.94–0.98 decreases and a significant peak of turbulence amplitude appears at *r*_eff_/*a*_99_ > 0.98. This data clearly shows that the turbulence moves outward and is exhausted to the scrape off layer. It should be noted that the increase of turbulence amplitude lasts only a few hundred *μ* sec and is much shorter than the time period of disappearance of large positive radial electric field as seen in Fig. 4c. Therefore, the abrupt increase of turbulence amplitude at *r*_eff_/*a*_99_ > 0.98 is not due to the change in radial electric field shear but to the outward movement of the turbulence cloud to the plasma edge. The sudden collapse after the ‘tongue’ event causes the outward shift of turbulence cloud as well as outward drift of trapped ions. This observation demonstrates that the magnitude of the turbulence is not determined by the local parameter such as temperature and pressure gradient. The turbulence cloud can be directly affected and transferred outward by this collapse. The causal relation between outward drift of trapped ion and transfer of turbulence cloud is open to question, and will be future work.

## Discussion

The outward velocity of displacement at *r*_eff_/*a*_99_ = 0.8 is ~200 m/s as seen in Fig. 2e. If this motion is interpreted to be driven by quasi-electrostatic perturbation field, the poloidal electric field causing this *E* × *B* motion is 300 V/m because the local toroidal magnetic field at this position is 1.5 T. As is explained in the following, we have a preliminary conclusion that the motion is due to *E* × *B* motion rather than the MHD perturbation. The magnitude of the displacement of the plasma, *ξ*, at the ‘tongue’ phase is much larger than that during the rotating mode, although the magnitude of perturbation of poloidal field, 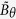, at the ‘tongue’ phase is comparable to that during the rotating mode, as seen in Fig. 1a,e. There are significant differences in the ratio of *ξ*/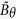 by the order of magnitude between the ‘tongue’ phase and the rotation mode phase. Because the ratio of *ξ*/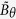 in quasi-electrostatic (QE) perturbations should be much larger that that in MHD perturbation, *ξ*/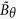
(QE) ≫ *ξ*/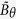
(MHD)^26^, these results strongly suggest that the perturbation at the ‘tongue’ phase is quasi-electrostatic (QE) perturbations, while the perturbation during the rotation mode is MHD perturbation.

The origin of the abrupt large scale motion in a ‘tongue’ phase can be interpreted as the *E* × *B* motion by the perturbation of the poloidal electric field (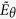 ~ 300 V/m). The magnetic perturbation observed is considered to be induced by finite-*β* effect^27^. If the plasma motion were frozen in the magnetic field (as MHD modes), the short parallel wavenumber of ‘tongue’ form demands tremendously large magnetic perturbation far beyond the measured 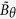. Since the time scale of the growth rate of this MHD activity is even less than one period of oscillations of magnetic field perturbation. The orbit drift of trapped ions is strongly affected by the radial electric field in toroidal plasmas. Therefore, the abrupt change in the radial electric field after the ‘tongue’ event would be a most possible mechanism causing the distortion of Maxwell-Boltzmann distribution of ions due to the outward drift of the trapped ions orbit.

There is a similarity of the ‘tongue’ event in other collapse events such as Edge-Localized mode (ELM) in tokamak plasmas^2^ and solar flare^5^,^28^. The fingerlike perturbation structure in the contour of electron temperature which starts to develops a few hundred *μ*s before the crash is localized both in toroidal/poloidal and radial direction. This fingerlike perturbation also extends radially before the crash, which is quite similar to the characteristics of ‘tongue’ shown in Fig. 2. The question regarding the trigger mechanism of solar flares (how and why the reconnection starts as an explosive process in flare event.) still remains open. The two-step magnetic reconnection model was proposed to explain the explosive property of the flare event. In this model, the reversal of magnetic shear causes a series of magnetic reconnections (second step of reconnection) and results in a large-scale eruption. There is a similarity of the transition of symmetry in magnetic field perturbation at the ‘tongue’ event to the reversal of magnetic shear which causes the large-scale eruption of the magnetic arcade in the solar flare. The ion cyclotron emission (ICE) driven primarily by velocity space gradients in non-Maxwellian energetic ion distributions are observed in fusion, space and astrophysical plasmas^29^.

In summary, the tongue of the magnetic field is clearly observed in the low density plasma with significant energetic ions injected by neutral beam. This is a new trigger mechanism of MHD burst different from the conventional picture where the instability of the MHD mode grows. A novel route, transition of symmetry, to trigger the onset of collapse of electron energy and MHD burst is identified. This mechanism is different from the conventional picture where the unstable MHD mode grows. The distortion of Maxwell-Boltzmann distribution of epithermal ions is observed for the first time in the plasma after the ‘tongue’ event. The mechanism for this distortion is rapid disappearance of radial electric field which suppresses the orbit loss of trapped ions in the plasma. There is no robust theory to explain the observed signal, solar flare and ELM’s, because the paths of the onsets have not been precisely observed in the past. Therefore, the discovery of the transition from mirror symmetric ‘tongue’ to helical symmetric mode with long wavelength along the magnetic field, provides a new and essential dynamical pattern, which should be looked for in future studies of abrupt events in plasmas.

## Methods

### Large Helical Device

The Large Helical Device (LHD) is a heliotron type device for magnetic confinement of high temperature plasmas with the magnetic field, B, of 2.7 T at the magnetic axis in the vacuum field, major, *R*_ax_, and effective minor radius *r*_eff_, of 3.6 m and 60–65 cm, respectively. In this experiment, the plasma density is 1−2 × 10^19^ m^−3^ and the central temperature is in the range of 2–4 keV. The LHD is equipped with three tangential neutral beams (NBs) in the opposite injection direction (two CCW and one CW) and two perpendicular neutral beams.

### Magnetic probe

The magnetic probes using advanced technology are installed inside the vacuum vessel of the LHD device to measure the perturbation of poloidal magnetic field at 6 toroidal locations (*ϕ* = 18°, 90°, 126°, 198°, 270°, 342°) and 14 poloidal locations (*θ* = 245°, 264°, 285°, 306°, 316°, 338°, 349°, 11°, 22°, 44°, 54°, 75°, 86°, 96°). The coupling area, which determined the sensitivity and frequency response, is ~300 cm^2^ in the rectangular shape with an electric shield. The frequency response of a sensing element is flat up to 200 kHz and the sensitivity is absolutely calibrated.

### RF radiation probe

Cyclotron motions of energetic ions in a plasma radiate the electromagnetic wave in a frequency range from tens to hundreds of MHz. The radiations from a plasma are measured with an RF radiation probe to study the behavior of energetic ions in LHD. The RF radiation probe on LHD consists of a dipole antenna in the vacuum vessel and spectrometers. The dipole antenna locates at the midplane of the vacuum vessel of the LHD device (*θ* ~ 0°). The filter-bank resolves the RF intensity signal into 14 frequency components from 70 to 2800 MHz and each intensity is detected in the spectrometer.

### Charge exchange spectroscopy

Charge exchange spectroscopy is a beam emission spectroscopy commonly used to measure ion temperature and toroidal and poloidal rotation velocity in the plasma using the charge exchange reaction between the neutral beam and fully ionized ions (carbon impurity in this experiment). Since the emission is localized at the intersection between the neutral beam and the line of sight, the charge exchange spectroscopy gives good spatial and time resolution. In this experiment, the spatial resolution is 2–4 cm and the time resolution is 1 ms in toroidal system and 5 ms in poloidal system. There are two charge exchange spectroscopy systems used in this experiment. One is the toroidal system for the measurements of ion distribution parallel to the magnetic field and the other is the poloidal system for the measurements of plasma flow perpendicular to the magnetic field to derive radial electric field profiles.

### Doppler reflectometer

The O-mode Doppler reflectometer gives both turbulence amplitude and phase velocity of the turbulence on the laboratory frame at the reflection point of the microwave. The phase velocity of the turbulence has Doppler shift by the plasma *E* × *B* rotation, and the change in radial electric field can be inferred from the change in phase velocity of the turbulence. The location of the reflection is determined by the cutoff density and the location of the measurements is inferred using the radial profiles of electron density measured with multi-channel FIR interferometer.

### Conditional reconstruction

Conditional reconstruction of data are used to improve the time resolution of the measurements when there are many repeated events. By taking the relative time difference of the measurements with respect to the events, the effective time resolution can be improved when the several events randomly occur in the steady state in the discharge.

## Additional Information

**How to cite this article**: Ida, K. *et al.* Abrupt onset of tongue deformation and phase space response of ions in magnetically-confined plasmas. *Sci. Rep.* **6**, 36217; doi: 10.1038/srep36217 (2016).

**Publisher’s note**: Springer Nature remains neutral with regard to jurisdictional claims in published maps and institutional affiliations.
